# Supplementary material for: Sorting at embryonic boundaries requires high heterotypic interfacial tension
Source: Nat Commun. 2017 Jul 31;8:157. doi: 10.1038/s41467-017-00146-x (PMC5537356; doi:10.1038/s41467-017-00146-x)
Supplement: Supplementary file 2 — Supplementary Software 1 [file 41467_2017_146_MOESM2_ESM.zip › PottsModel/SrcPottsModel/doc/index-files/index-8.html]

H-Index


JavaScript is disabled on your browser.


Skip navigation links


- Overview
- Package
- Class
- Use
- Tree
- Deprecated
- Index
- Help

- Prev Letter
- Next Letter

- Frames
- No Frames

- All Classes

A B C D E F G H I J K L M N O P R S T U V W 


## H

HalfLine - Class in model


HalfLine() - Constructor for class model.HalfLine


HalfLine(HalfLine) - Constructor for class model.HalfLine


HalfLine(Point, double) - Constructor for class model.HalfLine


HalfLine(Coordinates, Coordinates) - Constructor for class model.HalfLine


HalfLine(Point, Point) - Constructor for class model.HalfLine


hashCode() - Method in class gui.CellDisplay


hashCode() - Method in class model.Cell


hashCode() - Method in class model.Pixel


hasNext() - Method in class model.Lattice.PixelIterator


HBLStatistic - Class in engine


HBLStatistic(PottsEngine) - Constructor for class engine.HBLStatistic


HBLStatistic(PottsEngine, int) - Constructor for class engine.HBLStatistic


Hexagon - Class in gui
:   Flat topped hexagon.

Hexagon(int, int, int) - Constructor for class gui.Hexagon


Hexagon.AxialCoordinates - Class in gui


Hexagon.CubeCoordinates - Class in gui


Hexagon.OffsetCoordinates - Class in gui
:   Even-q vertical layout.

HexagonPixelDisplay - Class in gui


HexagonPixelDisplay(int, int, int, Color) - Constructor for class gui.HexagonPixelDisplay


HexagonPixelDisplay(int, int, int) - Constructor for class gui.HexagonPixelDisplay


HexagonPositionManager - Class in model


HexagonPositionManager(Lattice) - Constructor for class model.HexagonPositionManager


HexagonPositions - Static variable in interface model.IPositionManager


HMDStatistic - Class in engine
:   Homotypic Minimal Distance statistic.

HMDStatistic(PottsEngine, Cell.CellType[], int) - Constructor for class engine.HMDStatistic


HSLColor - Class in utils
:   The HSLColor class provides methods to manipulate HSL (Hue, Saturation
    Luminance) values to create a corresponding Color object using the RGB
    ColorSpace.

HSLColor(Color) - Constructor for class utils.HSLColor
:   Create a HSLColor object using an RGB Color object.

HSLColor(float, float, float) - Constructor for class utils.HSLColor
:   Create a HSLColor object using individual HSL values and a default
    alpha value of 1.0.

HSLColor(float, float, float, float) - Constructor for class utils.HSLColor
:   Create a HSLColor object using individual HSL values.

HSLColor(float[]) - Constructor for class utils.HSLColor
:   Create a HSLColor object using an an array containing the
    individual HSL values and with a default alpha value of 1.

HSLColor(float[], float) - Constructor for class utils.HSLColor
:   Create a HSLColor object using an an array containing the
    individual HSL values.

A B C D E F G H I J K L M N O P R S T U V W

Skip navigation links


- Overview
- Package
- Class
- Use
- Tree
- Deprecated
- Index
- Help

- Prev Letter
- Next Letter

- Frames
- No Frames

- All Classes
